# Combined transpetrosal approach for giant petroclival meningioma: 2-dimensional operative video

**DOI:** 10.3171/2022.1.FOCVID21248

**Published:** 2022-04-01

**Authors:** Vera Vigo, Karam Asmaro, Maximiliano A. Nuñez, Ahmed Moyheldin, Robert K. Jackler, Juan C. Fernandez-Miranda

**Affiliations:** 1 Department of Neurosurgery, Stanford University School of Medicine, Stanford, California; and; 2 Department of Otolaryngology–Head and Neck Surgery, Stanford University School of Medicine, Stanford, California

**Keywords:** petroclival meningioma, transpetrosal approach, middle fossa approach, anterior petrosectomy, retrosigmoid approach, retrolabyrinthine approach

## Abstract

Petroclival meningiomas are extremally challenging lesions due to their deep location and close relation to critical neurovascular structures. Several approaches have been described to achieve gross-total resection with low morbidity and mortality. In this 2-dimensional operative video, the authors show a simultaneous combined transpetrosal approach. The patient is a 44-year-old woman with an 8-month history of gait imbalance with evidence of a giant petroclival meningioma on neuroimaging. She underwent a combined middle fossa approach with anterior petrosectomy and retrosigmoid/retrolabyrinthine approach to achieve gross-total tumor resection. The postoperative course was characterized by trigeminal neuralgia, and neuroimaging showed gross-total resection of the tumor.

The video can be found here: https://stream.cadmore.media/r10.3171/2022.1.FOCVID21248

## Transcript

### 0:24 Clinical History.

This is the illustrative case for a 44-year-old female patient that presented with symptoms of hydrocephalus and difficulty swallowing. On imaging, she had this very large petroclival meningioma with severe brainstem compression and dilated ventricles. We can see the vascular supply coming from the tentorial branches and the severe compression of the midbrain.

### 0:53 Surgical Planning and Anatomy.

We plan a combined transpetrosal approach with a middle fossa dissection, posterior pedicle flap for reconstruction. We perform a large craniotomy with burr holes on both sides of the transverse sinus. These anatomy dissections illustrate the craniotomy that was performed exposing the transverse sinus, and then we’ll drill the mastoid. We will also perform our middle fossa approach with the goal of exposing Meckel’s cave and the trigeminal nerve branches in an external fashion. This allowed us to expose from the cavernous sinus back to the tentorium and Meckel’s cave and join these across the tentorium, to join the posterior fossa and middle fossa components of this tumor to maximize our tumor access.

### 1:41 Mastoidectomy.

We are now in surgery; the craniotomy has been performed already. Now we’re performing the mastoidectomy. This is a retrolabyrinthine approach with hearing preservation intention. The sigmoid sinus has been skeletonized. These are our neurotology team, Professor Jackler performing the approach.

### 2:00 Middle Fossa Dissection.

We’re now in the middle fossa; neurosurgery is doing this part. That’s V3, and we did identify the middle meningeal artery that we transect and coagulate. Now we peel the middle cranial fossa from anterior to posterior, identify the anterior wall of the cavernous sinus. Then back to the petrous apex to identify the greater petrosal superficial nerve and V3. Now we carefully dissect the trigeminal nerve back to Meckel’s cave.

### 2:24 Anterior Petrosectomy.

We then perform our anterior petrosectomy starting anterior and medial. We can also use an ultrasonic aspirator to facilitate this more narrow drilling.

### 2:39 Internal Auditory Canal Opening.

And now we go to the area of the internal auditory canal. Again, our neurotology colleagues are now opening the internal auditory canal and roofing it, and this provides extra exposure stimulating the facial nerve.

### 2:51 Dura and Tentorium Opening.

We’re now proceeding with the dura opening starting at the posterior cranial fossa, and then we’ll extend these into the cisterns, where we can see VII-VIII nerve complex and the tumor. In this case, we are fortunate to have a quite soft meningioma, which is very favorable. The tentorium has been now clipped at the level of the superior petrosal sinus, and then we can start our transection of the tentorium. We’ve opened the dura along the base of the medial temporal lobe or base of the middle cranial fossa. There is some tumor above the tentorium that we are now debulking. We can see the edge of the tentorium, the medial edge. We’re looking for the fourth nerve, but it’s been very displaced by the tumor, so it’s not at the usual location, as we will see later.

### 3:42 Tumor Removal and Meckel’s Cave Opening.

We nearly access the tumor that, as we said, is soft in consistency; however, it is quite vascular. We follow the trigeminal nerve and now we are opening Meckel’s cave here with a right-angle knife. I follow the dura along the trigeminal nerve to open the ring of Meckel’s cave, and this frees up the trigeminal nerve and allows me to access Meckel’s cave, and all the dura around Meckel’s cave has now been removed because it’s involved with tumor. This is the roof of Meckel’s cave that has been removed.

### 4:10 Tumor Removal: Posterior and Lateral Aspect.

And this widens my exposure to start working along the petroclival edge along the clivus, where I expect to find the sixth nerve, probably displaced anteriorly inferiorly by the tumor. I am working posteriorly along the trigeminal nerve between V and VII-VIII complex.

We can follow the trigeminal nerve all the way to the pons, and with careful microsurgical dissection technique we can respect the neurovascular structures. We’re working above V now, between V and III. And now we see all the lower nerves dissected inferiorly, VII, VIII, and then V, and there are different windows between cranial nerves that are giving us access to the deeper location of the tumor.

### 5:12 Tumor Removal Along VI Cranial Nerve.

You see that’s the tumor that is medial to V, and this is working between V and the medial temporal lobe. Cranial nerve III and VI are going to be identified medially. We are going to see cranial nerve VI, abducens nerve, displaced by the tumor. And, again, we progress with careful microsurgical dissection. We identify the sixth nerve origin at the brainstem. And this is great because we can then try to follow it, detach it from the tumor capsule, and follow it all the way to the dural entry point into Dorello’s canal.

### 5:50 Tumor Removal: Superior and Medial Aspect.

This portion of the tumor is the highest portion of the tumor toward the third cranial nerve and compressing the uncus. We finally see the basilar artery, we saw the anterior inferior cerebellar artery, and our next plane of dissection is going to be along the basilar artery and the brainstem, in particular the midbrain. We perform further debulking to facilitate mobilization of the tumor.

And again, this is the portion of the tumor that is highest; it requires some temporal lobe retraction. We can finally identify the third nerve medially displaced superior and medial; the fourth nerve is being identified finally, also displaced along with the third nerve superior and medial. We keep dissecting along the third nerve. We see the tumor attachment to the dura of the posterior clinoid.

### 6:47 Tumor Removal: Along the Basilar Artery and Its Perforators.

That’s again the basilar artery. We’ve carefully dissected this tumor behind this large perforating vessel. You will see the superior cerebellar artery partially encased in the tumor. We can debulk it and do careful microsurgical dissection to dissect the tumor and all these basilar artery branches. This is an area of surgery that we have to be extremely careful about because of the risk of pontine perforator stroke.

### 7:17 Final Tumor Removal.

We can finally see the midbrain, very compressed, flattened by the tumor. We can see the third nerve with a PCA above and, as we saw before, the SCA below. Now finally freed up from tumor, and that’s the sixth nerve, stimulates really well at the end of surgery, and near-complete tumor resection.

### 7:35 Reconstruction.

The reconstruction was performed with a large fat graft and with that posterior pedicle flap along the mastoid. It’s important to put a large fat graft to occupy all the empty space and prevent pseudomeningocele and CSF leaks postop.

### 7:48 Postoperative Course.

And as you see, a near-complete tumor resection with perhaps very residual risks along the posterior clinoid process. Thank you.

### 7:55 References^1–5^

## Disclosures

The authors report no conflict of interest concerning the materials or methods used in this study or the findings specified in this publication.

## Author Contributions

Primary surgeon: Fernandez-Miranda, Jackler. Assistant surgeon: Asmaro, Moyheldin. Editing and drafting the video and abstract: Vigo, Asmaro, Moyheldin. Critically revising the work: Fernandez-Miranda, Moyheldin. Reviewed submitted version of the work: Fernandez-Miranda, Vigo, Asmaro, Jackler. Approved the final version of the work on behalf of all authors: Fernandez-Miranda. Supervision: Fernandez-Miranda, Nuñez.
